# An International Comparison of the Effect of Policy Shifts to Organ Donation following Cardiocirculatory Death (DCD) on Donation Rates after Brain Death (DBD) and Transplantation Rates

**DOI:** 10.1371/journal.pone.0062010

**Published:** 2013-05-07

**Authors:** Aric Bendorf, Patrick J. Kelly, Ian H. Kerridge, Geoffrey W. McCaughan, Brian Myerson, Cameron Stewart, Bruce A. Pussell

**Affiliations:** 1 The Centre for Values, Ethics and the Law in Medicine, School of Public Health, Sydney Medical School, University of Sydney, Sydney, NSW, Australia; 2 Sydney School of Public Health, University of Sydney, Sydney, NSW, Australia; 3 Haematology Department, Royal North Shore Hospital, Sydney, NSW, Australia; 4 Royal Prince Alfred Hospital, University of Sydney, Sydney, NSW, Australia; 5 Centenary Research Institute, University of Sydney, Sydney, NSW, Australia; 6 The Centre for Health Governance, Law and Ethics, Sydney Law School, University of Sydney, Sydney, NSW, Australia; 7 Prince of Wales Clinical School, University of New South Wales, Sydney, NSW, Australia; 8 Department of Nephrology, Prince of Wales Hospital, Sydney, NSW, Australia; University of Colorado, United States of America

## Abstract

During the past decade an increasing number of countries have adopted policies that emphasize donation after cardiocirculatory death (DCD) in an attempt to address the widening gap between the demand for transplantable organs and the availability of organs from donation after brain death (DBD) donors. In order to examine how these policy shifts have affected overall deceased organ donor (DD) and DBD rates, we analyzed deceased donation rates from 82 countries from 2000–2010. On average, overall DD, DBD and DCD rates have increased over time, with the proportion of DCD increasing 0.3% per year (p = 0.01). Countries with higher DCD rates have, on average, lower DBD rates. For every one-per million population (pmp) increase in the DCD rate, the average DBD rate decreased by 1.02 pmp (95% CI: 0.73, 1.32; p<0.0001). We also found that the number of organs transplanted per donor was significantly lower in DCD when compared to DBD donors with 1.51 less transplants per DCD compared to DBD (95% CI: 1.23, 1.79; p<0.001). Whilst the results do not infer a causal relationship between increased DCD and decreased DBD rates, the significant correlation between higher DCD and lower DBD rates coupled with the reduced number of organs transplanted per DCD donor suggests that a national policy focus on DCD may lead to an overall reduction in the number of transplants performed.

## Introduction

Since the first successful kidney transplant at Harvard University more than a half century ago [1], organ transplantation has become the preferred modality for the treatment of organ failure and has led to improved survival, decreased morbidity, significantly improvement to quality of life and reduced healthcare costs. Improvements in both clinical outcomes and availability of organs for transplant worldwide now enable more than 100,000 patients annually to receive an organ that will either save, or dramatically improve their lives [2]. Unfortunately, in all countries that have organ transplant programs, the demand for transplanted organs far exceeds the number of organs available with current estimates showing that there are approximately 1.7 million people in need of a solid organ transplant worldwide [3].

Throughout the past 30 years, a broad range of organ donation policies have been adopted worldwide in an effort to address the ever-widening gap between organ supply and demand. These have included public outreach and education campaigns, pecuniary incentives to encourage donation, paired kidney exchange programs, utilization of organs from expanded criteria donors (ECD), and enactment of presumed consent (opt-out) legislation. In recent years, the policy change that has attracted the most attention and the most professional and bureaucratic support has been the shift to the adoption of policies that promote donation after cardiocirculatory death.

In most countries (especially those with a common law tradition) death is defined by reference either to irreversible cessation of the circulatory system (asystole) or irreversible cessation of brain function (brain death) [4], [5], [6], [7], [8]. Following the definition of irreversible cessation of brain function as death by the Harvard Ad Hoc Committee in 1968 and the dissemination of clinical criteria and tests for the diagnosis of brain death, donation after brain death (DBD) became the established mechanism of organ donation–dramatically increasing the number of donor organs, increasing the number of viable organs that could be recovered from donors, improving transplant outcomes and extending the indications for transplantation.

While most countries rely upon DBD for the vast majority of organs retrieved from deceased donors, in certain situations, some organs can be recovered from patients whose hearts have recently stopped beating. This procedure, which is known as donation after cardiocirculatory death (DCD), involves careful coordination of the time of asystole with the commencement of organ retrieval. And while transplant outcomes for DCD livers are generally poorer than those recovered from DBD donors [9], DCD kidneys show similar graft survival rates to DBD [10] and DCD lung graft survival rates may eclipse those of DBD [11]. Although complex, and at times controversial [12], [13], [14], DCD provides an additional pathway for obtaining much needed donor organs.

In an attempt to define standardized methodologies appropriate for DCDs, the First International Workshop on DCD held in Maastricht in 1995 defined four categories of DCD [15]. These are:

Category I: Dead on Arrival (Uncontrolled (uDCD))

Category II: Unsuccessful Resuscitation (Uncontrolled (uDCD))

Category III: Awaiting Cardiac Arrest (Controlled (cDCD))

Category IV: Cardiac Arrest While Brain Dead (Controlled (cDCD))

While suggestions have been made that many Category III DCD donors might become brain dead were they allowed to progress [16], [17], Category III DCD is, by far, the most widely practiced [18].

In the last 10 years there has been a marked worldwide increase in the number of countries that have adopted policies promoting DCD. A recent study, prepared on behalf of European Committee on Organ Transplantation, identified 10 countries in Europe that have adopted or are in the process of adopting policies to promote the use of DCD [18]. The United States and Canada have both vigorously pursued DCD policies [19], [20], [21], and in Latin America, DCD donation has also been reported in Bolivia, Brazil and Colombia. In Australia, the Commonwealth Government recently allocated large amounts of funding [22] to support strategies aimed at improving its consistently low deceased organ donation rates, including the adoption of policies that promote DCD [23].

While it has generally been assumed that DCD provides an effective pathway toward increasing donation rates, the impact that DCD has had on deceased organ transplantation rates and DBD rates worldwide has generally not been quantified. In this study, we analyzed deceased donation (DD) rates from 2000–2010 for 82 countries that have reported results to international organ donation bodies to determine what effect policy shifts to increase DCD rates have had on deceased organ transplantation and DBD rates.

## Methods

Rates for deceased solid organ donation and transplantation, DBD and DCD (both uncontrolled DCD (uDCD) and controlled (cDCD)) were collected from: the Agence de la Biomédecine, the Council of Europe, Eurotransplant, the International Registry of Donation and Transplantation (IRODaT), Nederlandse Transplantatie Stichting, the NHS Blood and Transplant Organisation, the Organización Nacional de Trasplantes (ONT) and the United Network of Organ Sharing (UNOS)/US Organ Procurement and Transplantation Network (OPTN), for countries that reported organ transplantation activity for the period of 2000–2010 (**Appendix S1**).

Countries were categorized into three groups, according to the number of deceased donations recorded annually. Group One were defined as those countries where the DD rate was at least 20 per million population (pmp) per year for at least five years between 2000 and 2010 inclusive. Group Two countries were those countries with a DD rate greater than or equal to 10 but less than 20 pmp per year for at least five years and Group Three were all other countries with less than 10 DD pmp per year for at least five years. Countries that reported deceased donation rates which never exceeded 0.1 DD pmp were excluded from analysis.

Linear mixed models were fitted to:

1. DBD rate pmp, with (a) DCD rate pmp as an explanatory variable; (b) cDCD rate pmp as an explanatory variable; (c) uDCD rate pmp an explanatory variable;
2. Organ Transplant rate pmp, with DBD and DCD rate as an explanatory variables;
3. Number of transplanted organs, with donor type (DBD or DCD) as an explanatory variable.

To account for the repeated measurements each model included year as a covariate with a random intercept and slope for each country [24]. The country Group was included as a categorical variable in all the models, with an interaction with year, to allow for different trends over time.

Results were considered statistically significant if p<0.05. Model checking was conducted by examining predicted values with observed and residual plots. For plots, a line of the predicted values was generated using Lowess smoothing [25]. All analyses and plots were conducted in Stata**™** 12 (StataCorp LP, College Station, TX USA).

## Results

Eighty-two (82) countries were identified as reporting any organ transplantation activity for the period of 2000–2010 and were included in our study. These countries, by geographic region, were:

Africa –5 countries (Algeria, Libya, Morocco, Tunisia, South Africa); Asia –10 countries (Bangladesh, Brunei, Hong Kong SARC, Japan, Malaysia, Pakistan, Philippines, Singapore, South Korea, Taiwan); Europe –35 countries (Austria, Belgium, Bulgaria, Croatia, Cyprus, Czech Republic, Denmark, Estonia, Finland, France, Georgia, Germany, Greece, Hungary, Iceland, Ireland, Italy, Latvia, Lithuania, Luxembourg, Malta, Moldova, Netherlands, Norway, Poland, Portugal, Romania, Russia, Slovakia, Slovenia, Spain, Sweden, Switzerland, Ukraine, United Kingdom); Latin America –18 countries (Argentina, Bolivia, Brazil, Chile, Colombia, Costa Rica, Cuba, Dominican Republic, Ecuador, El Salvador, Guatemala, Mexico, Panama, Paraguay, Peru, Trinidad and Tobago, Uruguay, Venezuela); Middle East –10 countries (Bahrain, Egypt, Iran, Israel, Jordan, Kuwait, Lebanon, Qatar, Saudi Arabia, Turkey); North America –2 countries (Canada, USA); and, Oceania –2 countries (Australia and New Zealand).

Of the 82 countries, 10 reported DD rates less than 0.1 pmp (Algeria, Bangladesh, Brunei, Egypt, El Salvador, Georgia, Libya, Morocco, Pakistan and Philippines) and were excluded from analysis. Thirty-one (31) of these 82 countries reported DCD donations (**Table 1**).

**Table 1:** Listing of Countries Utilizing DCD and Relevant Maastricht Categories (Adapted from Dominguez-Gil B, Haase-Kromwijk B, Van Leiden H, Neuberger J, Coene L, et al.) [18].

Country	Policy Shift to Focus on DCD	2010 DCD >10% of DD?	Primary Maastricht Category of DCD Donors
Australia	2004	Y	III
Austria	1994	N	II
Belgium	1994	Y	II, III
Canada	2006	N (9.8% in 2010)	III
Colombia	2009	Y	II
Czech Republic	1972	N	III
France	2006	N	I
Israel	2004	N	IV
Italy	2005	N	II
Japan	The Organ Transplant Act in 1997 made DBD legal in Japan. Even though it was revised in 2009 to encourage DBD, most donations continue to be DCD.	Y	II, III
Latvia	1992	Y	III
The Netherlands	1981	Y	II, III
New Zealand	2008	N	III
Russia	2008	Y	II, IV
Spain	1994	N	I
Switzerland	1993	N	III
UK	1989	Y	III
USA	1993	Y	III

In addition to the countries listed above, the following countries have reported occasional, very low rates of DCD donation activity since 2000: Algeria, Bolivia, Brazil, Croatia, Hong Kong SARC, Lebanon, Pakistan, Romania, Saudi Arabia, Singapore, South Korea, Turkey and Ukraine.

### Observed Trends in Group One Countries (≥20 DD pmp Per Year)

Group One included the following seven countries (listed in order of highest to lowest DD rates): Spain, Portugal, USA, Belgium, Austria, France and Italy. Of the countries that reported DCD donations in this group, Italy reported the lowest DCD rates at <1% of total DD, and Belgium reported the highest (21.71% of total DD in 2009). Portugal had no reported DCD donations. Spain and France utilized only uDCD.

The subset of these eight Group One countries that reported the highest sustained DD rates (≥25 DD pmp) consisted of three countries: Spain, Portugal and the USA. Of these three highest performing countries, only the USA had a DCD rate of more than 10% of overall DD (11.84% in 2010).

Of the entire Group One (≥20 DD pmp) cohort, only Belgium had a DCD rate above 12%. Much of the growth in Belgium’s DCD rate that occurred in the past 5 years correlated with a decrease in its DBD rates (**Appendix S2**).

With the exception of Belgium and Austria, all Group One countries maintained or showed a trend to increasing overall DD rates 2000–2010. In all cases, the increases in DD were due to sustained or increased donation after brain death, not donation after cardiocirculatory death (**Appendix S2**). Overall, there was a very strong relationship between high rates of DD and high rates of DBD.

### Observed Trends in Group Two Countries (<20 but ≥10 DD pmp Per Year)

Group Two included the following 26 countries (listed in order of highest to lowest DD rates): Norway, Croatia, Malta, Ireland, Estonia, Slovenia, Czech Republic, Finland, Hungary, Uruguay, Latvia, Cuba, UK, Germany, Argentina, Slovak Republic, Australia, Netherlands, Canada, Denmark, Sweden, Switzerland, Lithuania, Luxembourg, Poland and Costa Rica. Of these, the Czech Republic, Latvia, UK, Switzerland, Australia, Netherlands, and Canada reported DCD activity of ≥1 DCD pmp during one or more years of the time period studied. Norway, Croatia, Malta, Ireland, Estonia and Slovenia showed the highest overall DD rates, with sustained rates of ≥18 DD pmp. None of these countries reported DCD rates during the time period analyzed.

Amongst Group Two countries, as a percentage of total DD, Latvia reported the highest DCD percentage rate (53.8% in 2002) with the Netherlands reporting the highest sustained percentage with 37% during the time period 2000–2010 (**Appendix S3**). During this same period, the overall DD rate in the Netherlands remained largely stable between 12–13 DD pmp. The stasis of the Netherland’s DD rate was associated with a decline in DBD and an increase in DCD. The UK, Australia and (most recently) Canada all demonstrated increasing DCD growth rates coupled with static (Australia) or declining DBD rates (UK and Canada) during this period (**Appendix S3**).

Of the countries in Group Two that reported DCD donations, in both proportional and absolute terms, the Czech Republic reported the lowest DCD rates, with DCD rates never surpassing 0.3 pmp (or 1.5% of its total DD).

With the exception of Latvia (between 2004–2007), none of the countries in Group Two that reported DCD activity of ≥2% of total donors achieved sustained DD total rates >17 DD pmp. Other than Australia, all Group Two countries that reported DCD rates of ≥2% of total DD showed reductions in overall DD rates over the time period studied (**Appendix S3**).

### Observed Trends in Group Three Countries (<10 DD pmp Per Year)

Group Three included the following 39 countries (listed in order of highest to lowest DD rates): Iceland, Cyprus, Colombia, Israel, New Zealand, Chile, Brazil, Greece, Taiwan, Hong Kong SARC, Singapore, Saudi Arabia, Qatar, Kuwait, Panama, Turkey, South Africa, Iran, Venezuela, Mexico, Bahrain, Romania, Peru, South Korea, Russia, Bulgaria, Bolivia, Ecuador, Lebanon, Paraguay, Tunisia, Japan, Moldova, Malaysia, Dominican Republic, Ukraine, Guatemala, Trinidad & Tobago and Jordan. Of these, Colombia, Hong Kong, Israel, New Zealand, Singapore, Saudi Arabia, South Korea, Russia and Japan reported DCD activity. With a peak of 1.40 DCD pmp, Russia showed the highest level of DCD in all Group Three countries with Japan showing the second highest level at 0.80 DCD pmp. Despite maintaining relatively low DD rates that never exceeded 1.0 DD pmp, as a percentage of total DD donors, Japan showed the highest percentage rate of DCD donors of any country in our study with a DCD rate of between 66–100% of DD during the study period (**Appendix S4**).

### Trends Over Time (All Countries)

**Figure 1:**
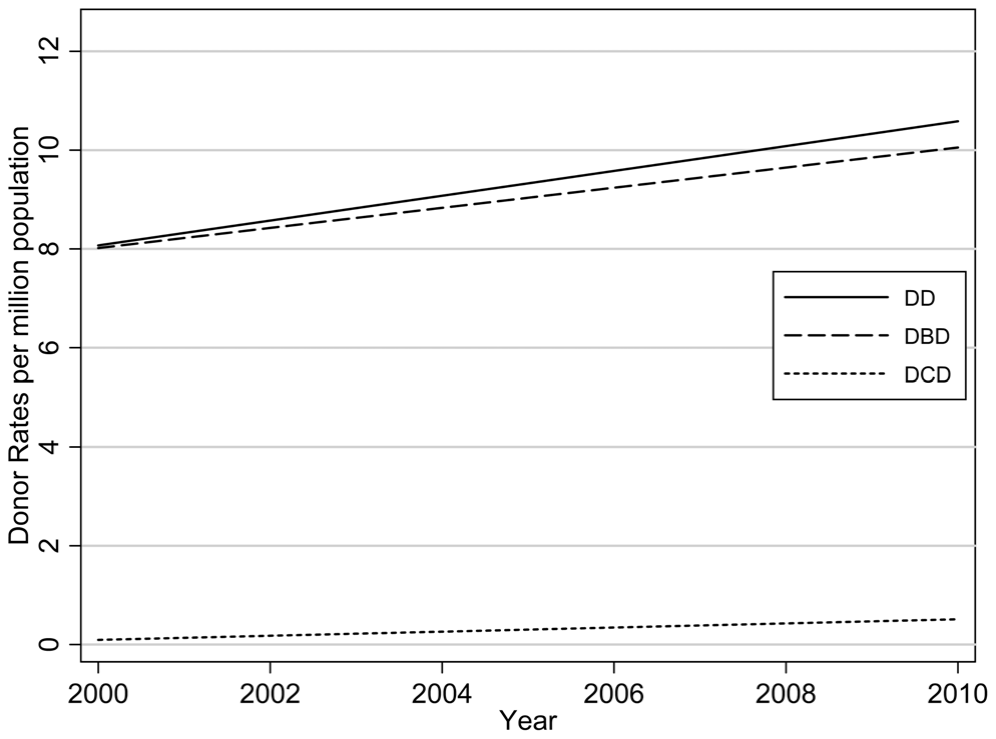
Overall average Deceased Donors (DD), Donation after Brain Death (DBD) and Donation after Cardiocirculatory Death (DCD) rates over time. Averages have been calculated from a linear mixed model, with random intercept and slope for country.

**Figure 2:**
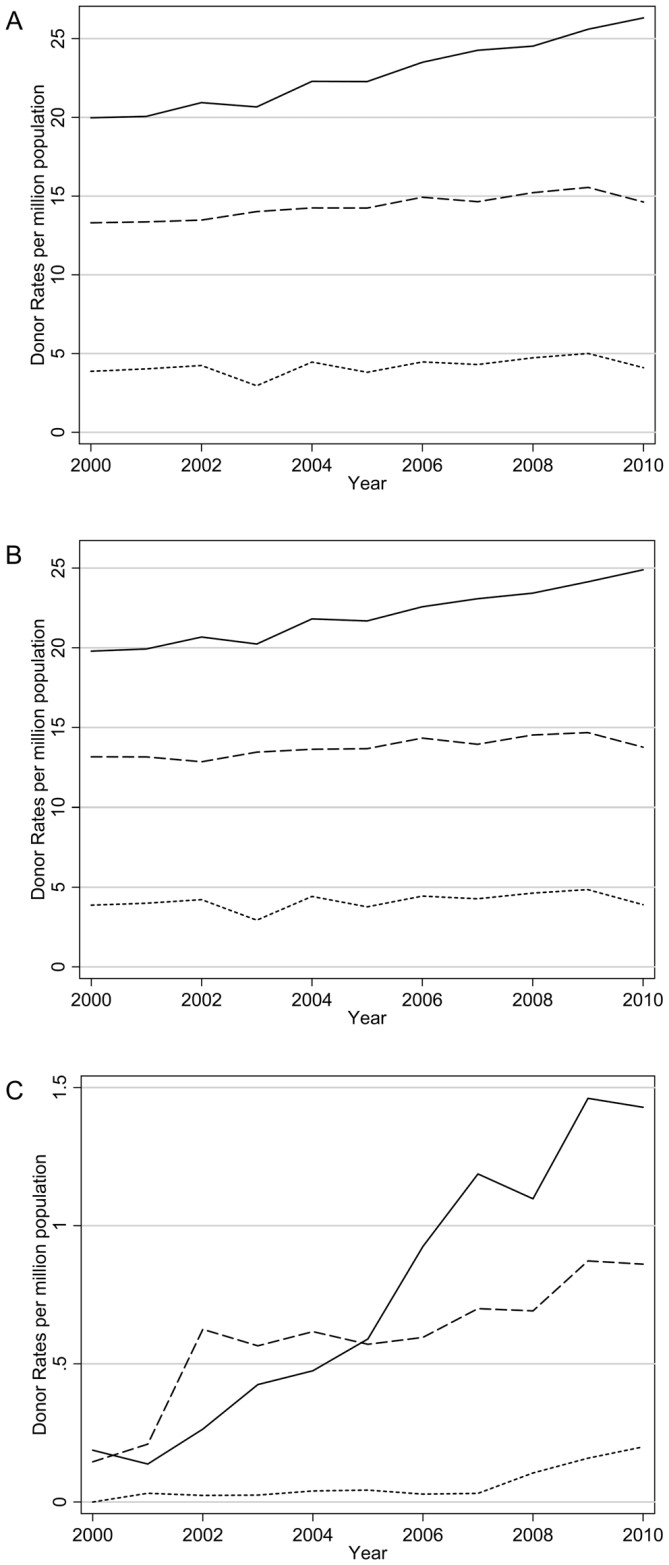
Average (a) Deceased Donors (DD), (b) Donation after Brain Death (DBD) and (c) Donation after Cardiocirculatory Death (DCD) rates over time by Group One (solid line), Group Two (dashed line) and Group Three (dotted line) countries.

There was a very wide range and high degree of variability between the DD, DCD, DBD and transplantation rates between the 82 countries. **Figure 1** shows the overall average DD, DBD and DCD rates over time. By definition, the overall DD rate is equal to the DBD rate plus the DCD rate. DCD makes a relatively small contribution to the DD rate, but the proportion is increasing over time, on average 0.3% increase per year (p = 0.01). The average DD, DBD and DCD rates all increased over time by Group (**Figure 2**), but with differing slopes. Over the period, the average DD, DBD and DCD rates increased most for Group One countries and the least for Group Three.

### DBD versus DCD Rates

**Figure 3:**
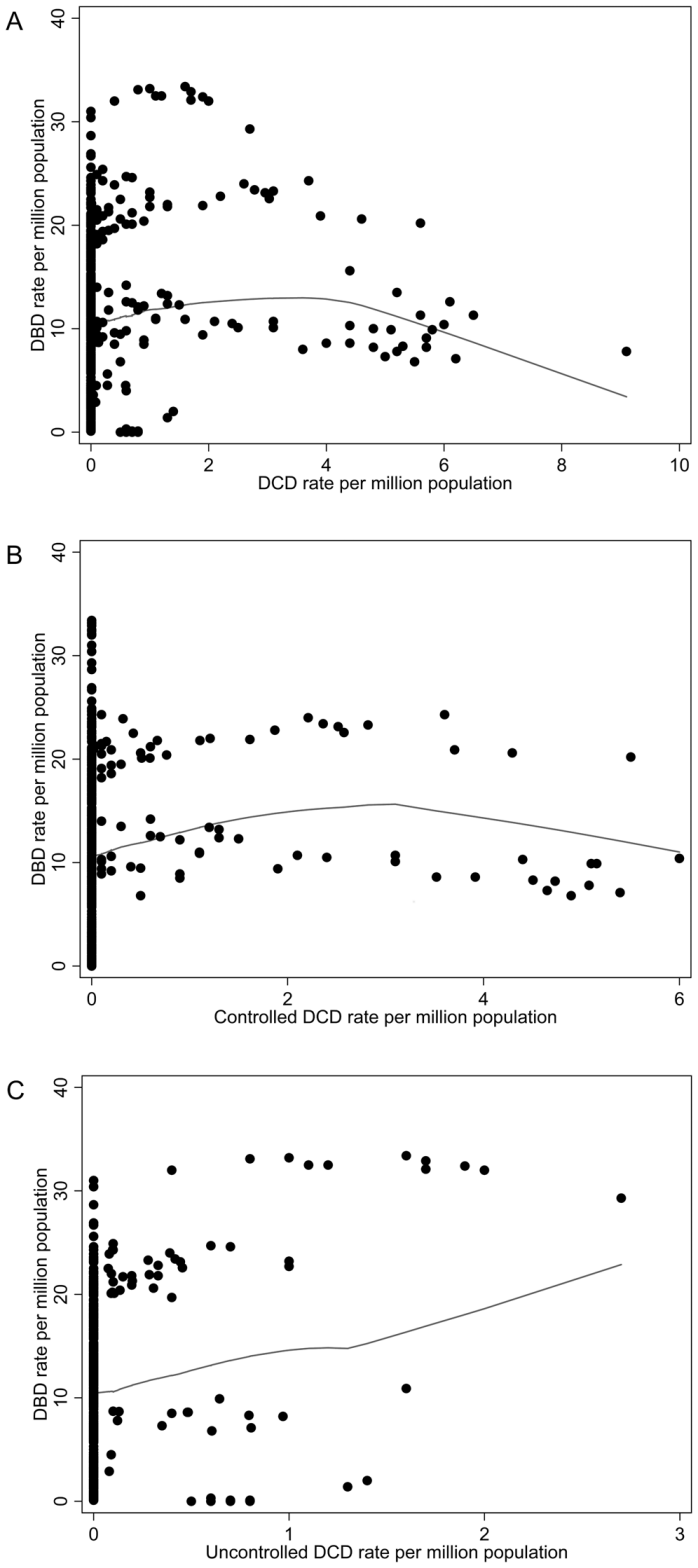
Donation after Brain Death (DBD) rates vs (a) Donation after Cardiocirculatory Death (DCD) rate (b) controlled Donation after Cardiocirculatory Death (cDCD) rate and (c) uncontrolled Donation after Cardiocirculatory Death (uDCD) rate. Solid lines are the Lowess curve of the predicted values from the fitted model.

**Figure 3** shows the DBD rates versus (**a**) overall DCD rates and by (**b**) cDCD and (**c**) uDCD rates respectively. For every unit pmp increase in the DCD rate, the average DBD rate decreased by 1.02 pmp (95% CI: 0.73, 1.32; p<0.0001). Similarly, for every unit pmp increase in the cDCD rate, the average DBD rate decreased by 0.84 (95% CI: 0.54, 1.24; p = 0.0014). And for every unit pmp increase in the uDCD rate, the average DBD rate decreased by 0.50 (95% CI: −1.62, 0.62; p = 0.438).

### Number of Organs Transplanted

**Figure 4:**
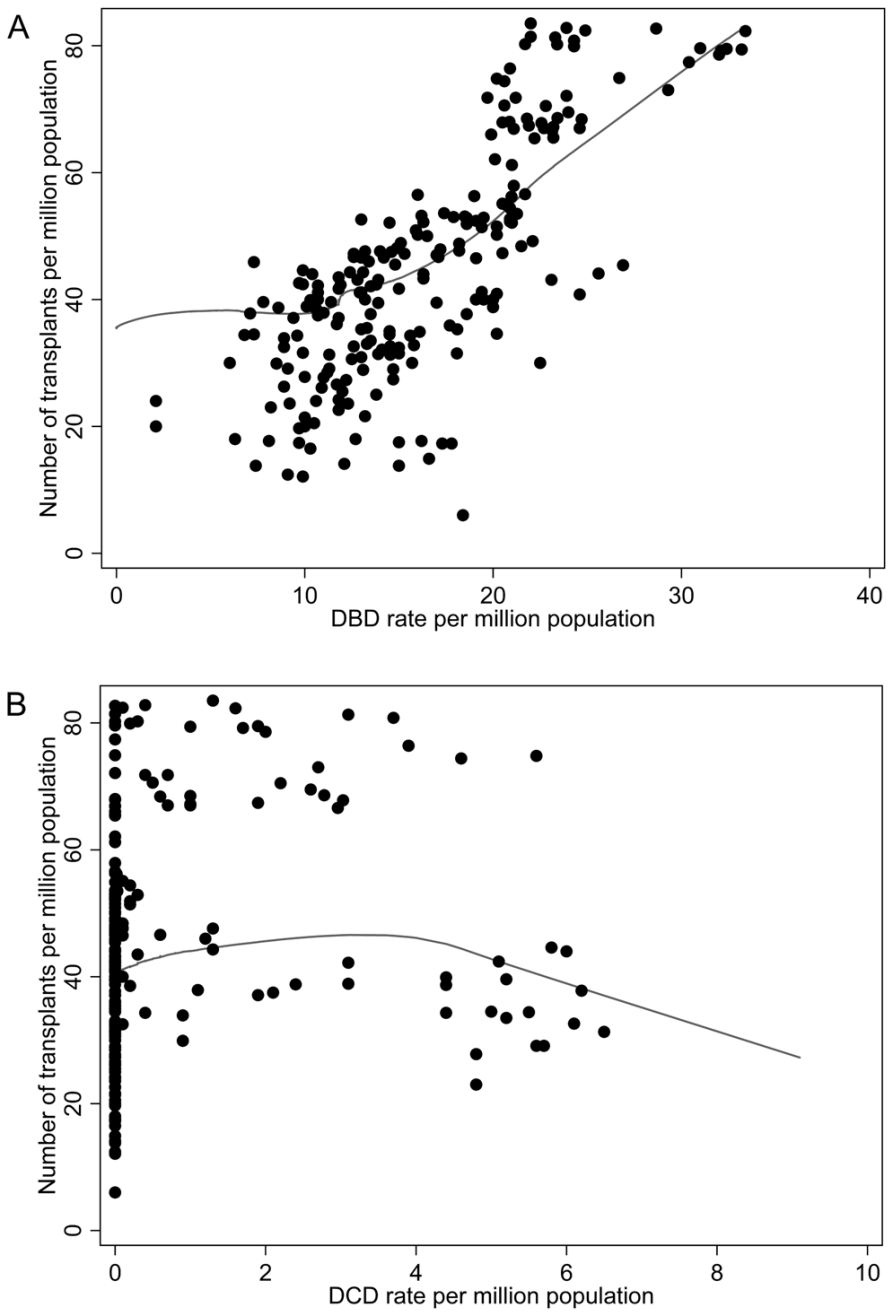
Transplant Rate by (a) Donation after Brain Death (DBD) rate and (b) Donation after Cardiocirculatory Death (DCD) rate. Solid lines are the Lowess curve of the predicted values from the fitted model.

**Figure 5:**
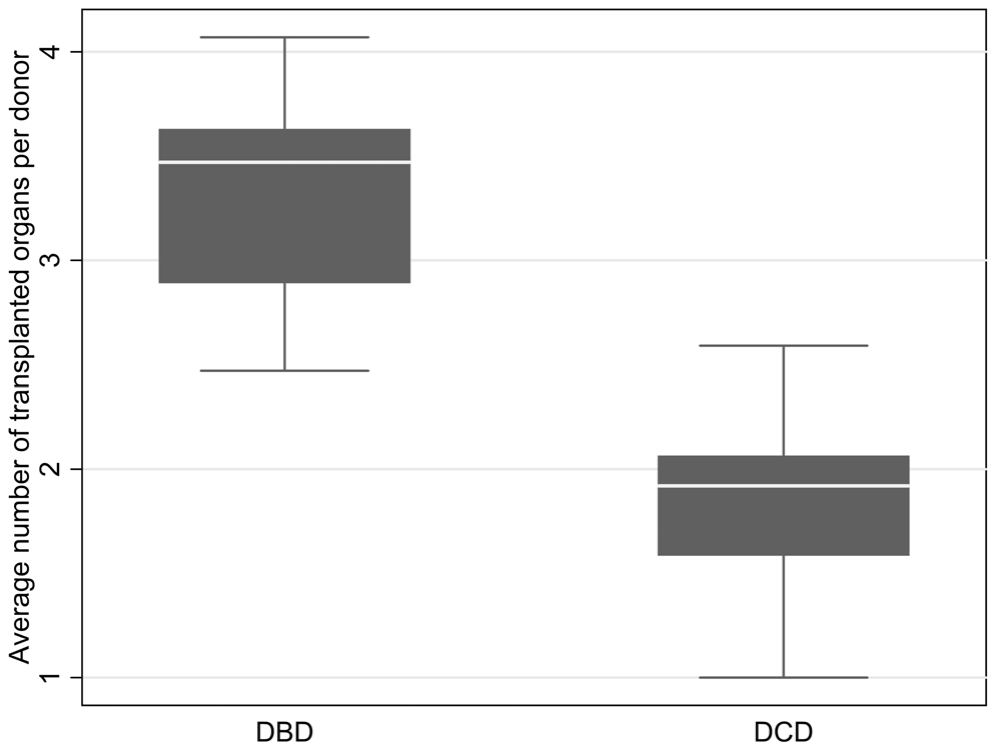
Boxplots of Average Number of Transplanted Organs per DBD and DCD Donor. Mean difference for number of transplants by donor type = 1.51 (95% CI: 1.42,1.60, p<0.001).

**Figure 4** shows the transplant rates versus DBD (**Figure 4a**) and DCD rates (**Figure 4b**). The estimated increase in the average number of transplants pmp is 1.30 for every unit pmp increase in the DBD rate (95% CI: 0.56, 2.05; p<0.001). The estimated increase in the average number of transplants pmp for every unit pmp increase in DCD rate pmp is 1.15, (95% CI: 0.32, 1.98; p = 0.007). **Figure 5** shows the mean difference between number of transplants by DBD and DCD donor type is 1.51 (95% CI: 1.23, 1.79; p<0.001).

## Discussion

Policies that emphasize DCD may be adopted for many reasons–principally to capture the increased number of people surviving initial trauma (that previously may have led to BD) and as a political strategy to increase the donor pool. While our data does not allow us to distinguish the reasons behind increasing DCD rates, our results reveal that higher rates of donation after cardiocirculatory death are associated with lower DBD rates, reduced organ transplantation rates and that high DCD rates are most frequently linked with the lower-middle range deceased donation rates of Group Two countries. High DBD rates and not high DCD rates appear to be the determining factor of the high DD rates (>20 pmp) observed in Group One countries. As this is an ecological study, we cannot say that the adoption of DCD policies are the *cause* of lower DBD rates as reasons for the lower DBD rates cannot be linked directly to DCD rates. Nevertheless, while there may be a number of factors contributing to reductions in DBD rates in countries that increasingly focus on DCD [26], our study provides support for the contention that DCD donors are, at least in part, being sourced from potential DBD donors [27].

Other factors, including increasing levels of public safety, improvements in resuscitation technology, and improved neurological treatment protocols for acute brain injury may also explain reductions in DBD rates [28]. It is worth noting, however, that recent global analysis of national stroke and traffic fatality rates (the most frequent causes of deaths that lead to organ donation) demonstrates clearly that the rapidly declining rates for these types of death in Group One (≥20 DD pmp) countries over the past two decades, has not negatively impacted upon these countries’ high DBD rates [29]. It is also noteworthy that several of the countries with the highest DBD rates had amongst the lowest stroke and traffic fatality rates [29].

Our results also show a significant differential between the number of organs transplanted from each donor type. While we did not examine potential reasons for the reduced number of organs transplanted in DCD, recent research has shown that the number of liver retrievals is particularly affected by DCD [30]. This, in itself, does not suggest that DCD policies should be curtailed or abandoned. It does, however, illustrate a potential limitation of DCD upon organ transplantation and the need for ongoing research to improve organ and patient outcomes following DCD.

There are a number of potential limitations to this study, due primarily to two factors. First, as mentioned previously, because this is an ecological study, causal relationships cannot be inferred from our results. Second, completing the data set for such a large number of countries required us to draw from multiple data sources (**Appendix S1**). Because different organizations and registries rely upon different metrics, standards and personnel for data collection and reporting, this may have introduced variation into the data set. Nevertheless, this study represents the most comprehensive international analysis of DCD yet undertaken.

While improvements in organ and patient outcomes – both from DCD and DBD donors – will undoubtedly continue, our results show that, per donor, DCD reduces the number of transplants performed when compared to DBD. The risk, therefore, is that if policies that promote DCD come at the expense of DBD, they may come at a high cost and may not be the most appropriate long-term strategy for improving organ transplantation rates. While DCD policies undoubtedly have an important role to play in comprehensive national frameworks for organ donation, it is impossible to avoid the conclusion that efforts aimed at significantly increasing transplantation rates ultimately require the implementation of policies that increase the identification of more DBD donors [31] and a serious reconsideration of approaches to the “active management” of dying and the use of technology at the end of life.

## Supporting Information

Appendix S1 Data Sources. (DOCX)

Appendix S2 Group One Countries. (DOCX)

Appendix S3 Group Two Countries. (DOCX)

Appendix S4 Group Three Countries. (DOCX)
