# Supplementary material for: An International Comparison of the Effect of Policy Shifts to Organ Donation following Cardiocirculatory Death (DCD) on Donation Rates after Brain Death (DBD) and Transplantation Rates
Source: PLoS One. 2013 May 7;8(5):e62010. doi: 10.1371/journal.pone.0062010 (PMC3647074; doi:10.1371/journal.pone.0062010)
Supplement: Appendix S1 — (DOCX) [file pone.0062010.s001.docx]

**Appendix S1**

**Data Sources**

| **Data Source** |  | **Available at:** |
| --- | --- | --- |
| Agence de la Biomédecine |  | [http://www.agence-medecine.fr/Tous-nos-rapports-et-etudes](http://www.agence-biomedecine.fr/Tous-nos-rapports-et-etudes)  [Last accessed on January 23, 2013] |
| Council of Europe |  | <http://www.ont.es/publicaciones/Paginas/Publicaciones.aspx>  [Last accessed on January 23, 2013] |
| Eurotransplant |  | <http://www.eurotransplant.org/cms/index.php?page=annual_reports>  [Last accessed on January 23, 2013] |
| IRODaT |  | <http://tpm.org.es>  [Last accessed on January 23, 2013] |
| Nederlandse Transplantatie Stichting |  | <http://www.transplantatiestichting.nl/professionals/cijfers-voor-professionals>  [Last accessed on January 23, 2013] |
| NHS Blood and Transplant Organisation |  | <http://www.organdonation.nhs.uk/statistics/transplant_activity_report/archive_activity_reports/pdf/ukt/activity_report_2010_11.pdf>  [Last accessed on January 23, 2013] |
| Organización Nacional de Trasplantes (ONT) |  | <http://www.ont.es/infesp/Paginas/Memorias.aspx>  [Last accessed on January 23, 2013] |
| UNOS/OPTN |  | <http://www.aopo.org/related-links-data-organ-donation-transplantation-a40>  [Last accessed on January 23, 2013] |
