# Supplementary material for: An International Comparison of the Effect of Policy Shifts to Organ Donation following Cardiocirculatory Death (DCD) on Donation Rates after Brain Death (DBD) and Transplantation Rates
Source: PLoS One. 2013 May 7;8(5):e62010. doi: 10.1371/journal.pone.0062010 (PMC3647074; doi:10.1371/journal.pone.0062010)
Supplement: Appendix S2 — (DOCX) [file pone.0062010.s002.docx]

**Appendix S2**

**Group One Countries (DD Rate ≥20 DD pmp per year) – DD, DBD & DCD Rates in pmp**

| **Country** | **2000** | **2001** | **2002** | **2003** | **2004** | **2005** | **2006** | **2007** | **2008** | **2009** | **2010** |
| --- | --- | --- | --- | --- | --- | --- | --- | --- | --- | --- | --- |
| Spain DD Rate | 33.90 | 32.40 | 33.60 | 33.70 | 34.60 | 35.00 | 33.80 | 34.30 | 34.20 | 34.00 | 32.00 |
| Spain DBD Rate | 33.10 | 32.00 | 32.50 | 32.50 | 32.90 | 33.40 | 32.10 | 32.40 | 33.20 | 32.00 | 29.30 |
| Spain DCD Rate | 0.80 | 0.40 | 1.10 | 1.20 | 1.70 | 1.60 | 1.70 | 1.90 | 1.00 | 2.00 | 2.70 |
| Portugal DD Rate | 19.40 | 20.20 | 21.70 | 19.00 | 22.10 | 19.00 | 20.10 | 23.90 | 26.70 | 31.00 | 30.40 |
| Portugal DBD Rate | 19.40 | 20.20 | 21.70 | 19.00 | 22.10 | 19.00 | 20.10 | 23.90 | 26.70 | 31.00 | 30.40 |
| Portugal DCD Rate | - | - | - | - | - | - | - | - | - | - | - |
| USA DD Rate | 23.00 | 20.70 | 20.80 | 21.30 | 23.10 | 23.80 | 25.00 | 26.60 | 26.20 | 26.10 | 25.60 |
| USA DBD Rate | 22.50 | 20.10 | 20.10 | 20.40 | 21.80 | 21.90 | 22.80 | 24.00 | 23.42 | 23.14 | 22.57 |
| USA DCD Rate | 0.50 | 0.60 | 0.70 | 0.90 | 1.30 | 1.90 | 2.20 | 2.60 | 2.78 | 2.96 | 3.03 |
| Belgium DD Rate | 25.60 | 21.50 | 21.60 | 23.30 | 21.10 | 22.80 | 26.40 | 28.00 | 24.80 | 25.80 | 25.20 |
| Belgium DBD Rate | 25.40 | 21.40 | 21.30 | 22.00 | 20.60 | 21.80 | 23.30 | 24.30 | 20.90 | 20.20 | 20.60 |
| Belgium DCD Rate | 0.20 | 0.10 | 0.30 | 1.30 | 0.50 | 1.00 | 3.10 | 3.70 | 3.90 | 5.60 | 4.60 |
| Austria DD Rate | 23.20 | 23.60 | 22.10 | 22.00 | 22.00 | 24.50 | 24.30 | 21.90 | 20.10 | 25.00 | 23.40 |
| Austria DBD Rate | 23.20 | 23.60 | 22.10 | 22.00 | 21.70 | 24.30 | 23.90 | 21.20 | 19.70 | 24.90 | 23.40 |
| Austria DCD Rate | - | - | - | - | 0.30 | 0.20 | 0.40 | 0.70 | 0.40 | 0.10 | 0.00 |
| France DD Rate | 16.90 | 17.80 | 20.30 | 18.60 | 21.00 | 22.20 | 23.20 | 25.30 | 25.30 | 24.20 | 23.70 |
| France DBD Rate | 16.90 | 17.80 | 20.30 | 18.60 | 21.00 | 22.20 | 23.20 | 24.70 | 24.60 | 23.20 | 22.70 |
| France DCD Rate | - | - | - | - | 0.00 | 0.00 | 0.00 | 0.60 | 0.70 | 1.00 | 1.00 |
| Italy DD Rate | 15.20 | 17.00 | 18.10 | 18.50 | 21.10 | 21.00 | 21.70 | 20.90 | 21.00 | 21.30 | 21.60 |
| Italy DBD Rate | 15.20 | 17.00 | 18.10 | 18.50 | 21.10 | 20.98 | 21.70 | 20.90 | 21.00 | 21.27 | 21.50 |
| Italy DCD Rate | - | - | - | - | 0.00 | 0.02 | 0.00 | 0.00 | 0.00 | 0.03 | 0.10 |
