# Supplementary material for: An International Comparison of the Effect of Policy Shifts to Organ Donation following Cardiocirculatory Death (DCD) on Donation Rates after Brain Death (DBD) and Transplantation Rates
Source: PLoS One. 2013 May 7;8(5):e62010. doi: 10.1371/journal.pone.0062010 (PMC3647074; doi:10.1371/journal.pone.0062010)
Supplement: Appendix S3 — (DOCX) [file pone.0062010.s003.docx]

**Appendix S3**

**Group Two Countries (<20 but ≥10 DD pmp per year) – DD, DBD & DCD Rates in pmp**

| **Country** | **2000** | **2001** | **2002** | **2003** | **2004** | **2005** | **2006** | **2007** | **2008** | **2009** | **2010** |
| --- | --- | --- | --- | --- | --- | --- | --- | --- | --- | --- | --- |
| Norway DD Rate | 17.50 | 14.30 | 13.60 | 18.90 | 19.50 | 16.50 | 16.20 | 19.90 | 20.50 | 21.10 | 20.87 |
| Norway DBD Rate | 17.50 | 14.30 | 13.60 | 18.90 | 19.50 | 16.50 | 16.20 | 19.90 | 20.50 | 21.10 | 20.87 |
| Norway DCD Rate | - | - | - | - | - | - | - | - | - | - | - |
| Croatia DD Rate | 2.60 | 7.30 | 9.30 | 8.90 | 13.30 | 9.90 | 13.50 | 13.20 | 17.90 | 17.40 | 28.66 |
| Croatia DBD Rate | 2.60 | 7.30 | 9.30 | 8.90 | 13.30 | 9.90 | 13.50 | 13.20 | 17.90 | 17.40 | 28.66 |
| Croatia DCD Rate | - | - | - | - | - | - | - | - | - | - | - |
| Malta DD Rate | 12.80 |  | 15.00 | 15.00 | 10.00 | 10.00 | 22.50 | 7.50 | 22.50 | 20.00 | 22.50 |
| Malta DBD Rate | 12.80 |  | 15.00 | 15.00 | 10.00 | 10.00 | 22.50 | 7.50 | 22.50 | 20.00 | 22.50 |
| Malta DCD Rate | 0.00 | - | - | - | - | - | - | - | - | - | - |
| Ireland DD Rate | 19.40 | 18.90 | 20.00 | 20.50 | 22.10 | 18.20 | 20.20 | 20.20 | 7.30 | 16.30 | 9.70 |
| Ireland DBD Rate | 19.40 | 18.90 | 20.00 | 20.50 | 22.10 | 18.20 | 20.20 | 20.20 | 7.30 | 16.30 | 9.70 |
| Ireland DCD Rate | - | - | - | - | - | - | - | - | - | - | - |
| Estonia DD Rate | - | - | 20.70 | 10.00 | 13.80 | 26.90 | 7.40 | 18.60 | 23.10 | 24.60 | 15.70 |
| Estonia DBD Rate | - | - | 20.70 | 10.00 | 13.80 | 26.90 | 7.40 | 18.60 | 23.10 | 24.60 | 15.70 |
| Estonia DCD Rate | - | - | - | - | - | - | - | - | - | - | - |
| Slovenia DD Rate | 11.00 | 11.50 | 18.40 | 14.00 | 18.00 | 10.50 | 15.00 | 12.00 | 19.50 | 17.00 | 20.20 |
| Slovenia DBD Rate | 11.00 | 11.50 | 18.40 | 14.00 | 18.00 | 10.50 | 15.00 | 12.00 | 19.50 | 17.00 | 20.20 |
| Slovenia DCD Rate | - | - | - | - | - | - | - | - | - | - | - |
| Czech Republic DD Rate | 17.00 | 16.70 | 13.80 | 18.30 | 20.60 | 19.80 | 18.80 | 21.10 | 19.20 | 19.10 | 19.60 |
| Czech Republic DBD Rate | 17.00 | 16.70 | 13.50 | 18.20 | 20.50 | 19.50 | 18.60 | 20.90 | 19.10 | 19.10 | 19.40 |
| Czech Republic DCD Rate | - | - | 0.30 | 0.10 | 0.10 | 0.30 | 0.20 | 0.20 | 0.10 | 0.00 | 0.20 |
| Finland DD Rate | 20.00 | 17.00 | 17.10 | 16.30 | 20.90 | 16.30 | 20.70 | 17.20 | 15.00 | 17.00 | 17.10 |
| Finland DBD Rate | 20.00 | 17.00 | 17.10 | 16.30 | 20.90 | 16.30 | 20.70 | 17.20 | 15.00 | 17.00 | 17.10 |
| Finland DCD Rate | - | - | - | - | - | - | - | - | - | - | - |
| Hungary DD Rate | 13.10 | 13.50 | 16.70 | 16.10 | 13.00 | 18.10 | 17.70 | 15.00 | 14.70 | 13.90 | 15.80 |
| Hungary DBD Rate | 13.10 | 13.50 | 16.70 | 16.10 | 13.00 | 18.10 | 17.70 | 15.00 | 14.70 | 13.90 | 15.80 |
| Hungary DCD Rate | - | - | - | - | - | - | - | - | - | - | - |
| Uruguay DD Rate | 10.40 | 11.50 | 14.70 | 16.10 | 18.10 | 20.00 | 25.60 | 18.08 | 19.09 | 19.40 | 14.54 |
| Uruguay DBD Rate | 10.40 | 11.50 | 14.70 | 16.10 | 18.10 | 20.00 | 25.60 | 18.08 | 19.09 | 19.40 | 14.54 |
| Uruguay DCD Rate | - | - | - | - | - | - | - | - | - | - | - |
| Latvia DD Rate | 17.30 | 24.30 | 16.90 | 16.90 | 17.80 | 20.00 | 18.70 | 18.70 | 13.00 | 14.80 | 14.80 |
| Latvia DBD Rate | 17.30 | 24.30 | 7.80 | 11.30 | 11.30 | 15.60 | 13.50 | 12.60 | 8.20 | 9.10 | 10.00 |
| Latvia DCD Rate | - | - | 9.10 | 5.60 | 6.50 | 4.40 | 5.20 | 6.10 | 4.80 | 5.70 | 4.80 |
| Cuba DD Rate | 8.60 | 12.50 | 19.90 | 17.30 | 16.20 | 9.10 | 15.00 | 17.80 | 16.60 | - | 9.90 |
| Cuba DBD Rate | 8.60 | 12.50 | 19.90 | 17.30 | 16.20 | 9.10 | 15.00 | 17.80 | 16.60 | - | 9.90 |
| Cuba DCD Rate | - | - | - | - | - | - | - | - | - | - | - |
| UK DD Rate | 13.20 | 13.20 | 13.10 | 12.00 | 13.80 | 12.80 | 12.90 | 13.20 | 14.70 | 15.00 | 16.40 |
| UK DBD Rate | 12.60 | 12.50 | 12.20 | 10.90 | 12.30 | 10.70 | 10.50 | 10.10 | 10.30 | 9.90 | 10.40 |
| UK DCD Rate | 0.60 | 0.70 | 0.90 | 1.10 | 1.50 | 2.10 | 2.40 | 3.10 | 4.40 | 5.10 | 6.00 |
| Germany DD Rate | 12.40 | 13.00 | 12.40 | 13.80 | 13.10 | 14.80 | 15.30 | 16.00 | 14.60 | 14.90 | 15.90 |
| Germany DBD Rate | 12.40 | 13.00 | 12.40 | 13.80 | 13.10 | 14.80 | 15.30 | 16.00 | 14.60 | 14.90 | 15.90 |
| Germany DCD Rate | - | - | - | - | - | - | - | - | - | - | - |
| Argentina DD Rate | 7.20 | 6.90 | 6.50 | 8.10 | 12.30 | 10.60 | 11.70 | 12.20 | 13.10 | 12.50 | 14.54 |
| Argentina DD Rate | 7.20 | 6.90 | 6.50 | 8.10 | 12.30 | 10.60 | 11.70 | 12.20 | 13.10 | 12.50 | 14.54 |
| Argentina DD Rate | - | - | - | - | - | - | - | - | - | - | - |
| Slovak Republic DD Rate | 9.20 | 10.50 | 6.80 | 8.50 | 10.10 | 11.80 | 11.80 | 20.20 | 14.50 | - | - |
| Slovak Republic DBD Rate | 9.20 | 10.50 | 6.80 | 8.50 | 10.10 | 11.80 | 11.80 | 20.20 | 14.50 | - | - |
| Slovak Republic DCD Rate | - | - | - | - | - | - | - | - | - | - | - |
| Australia DD Rate | 10.20 | 9.50 | 10.40 | 9.00 | 10.80 | 10.00 | 9.80 | 9.40 | 12.10 | 11.30 | 13.80 |
| Australia DBD Rate | 10.10 | 9.40 | 10.30 | 8.90 | 10.60 | 9.60 | 8.90 | 8.50 | 11.00 | 9.40 | 10.70 |
| Australia DCD Rate | 0.10 | 0.10 | 0.10 | 0.10 | 0.20 | 0.40 | 0.90 | 0.90 | 1.10 | 1.90 | 3.10 |
| Netherlands DD Rate | 12.60 | 11.60 | 12.60 | 13.60 | 13.90 | 13.30 | 12.30 | 15.70 | 12.30 | 13.00 | 13.00 |
| Netherlands DBD Rate | 10.10 | 8.00 | 8.60 | 8.30 | 8.20 | 7.10 | 6.80 | 9.90 | 7.30 | 7.80 | 8.60 |
| Netherlands DCD Rate | 2.50 | 3.60 | 4.00 | 5.30 | 5.70 | 6.20 | 5.50 | 5.80 | 5.00 | 5.20 | 4.40 |
| Canada DD Rate | 15.30 | 13.50 | 12.80 | 13.50 | 13.00 | 12.80 | 14.10 | 14.80 | 14.60 | 14.50 | 13.70 |
| Canada DBD Rate | 15.30 | 13.50 | 12.80 | 13.50 | 13.00 | 12.80 | 14.00 | 14.20 | 13.40 | 13.20 | 12.40 |
| Canada DCD Rate | 0.00 | 0.00 | 0.00 | 0.00 | 0.00 | 0.00 | 0.10 | 0.60 | 1.20 | 1.30 | 1.30 |
| Denmark DD Rate | 12.50 | 12.90 | 12.70 | 13.90 | 11.90 | 11.70 | 11.40 | 13.20 | 11.80 | 13.90 | 12.92 |
| Denmark DBD Rate | 12.50 | 12.90 | 12.70 | 13.90 | 11.90 | 11.70 | 11.40 | 13.20 | 11.80 | 13.90 | 12.92 |
| Denmark DCD Rate | - | - | - | - | - | - | - | - | - | - | - |
| Sweden DD Rate | 10.90 | 12.10 | 10.90 | 12.80 | 13.60 | 14.20 | 15.10 | 14.50 | 16.00 | 13.00 | 12.60 |
| Sweden DBD Rate | 10.90 | 12.10 | 10.90 | 12.80 | 13.60 | 14.20 | 15.10 | 14.50 | 16.00 | 13.00 | 12.60 |
| Sweden DCD Rate | - | - | - | - | - | - | - | - | - | - | - |
| Switzerland DD Rate | 14.00 | 13.10 | 10.40 | 12.90 | 12.60 | 12.10 | 10.70 | 10.80 | 11.80 | 13.00 | 12.60 |
| Switzerland DBD Rate | 14.00 | 13.10 | 9.80 | 12.10 | 11.80 | 11.80 | 10.70 | 10.70 | 11.80 | 13.00 | 12.60 |
| Switzerland DCD Rate | - | - | 0.60 | 0.80 | 0.80 | 0.30 | 0.00 | 0.10 |  | 0.00 | 0.00 |
| Lithuania DD Rate | - | - | 7.90 | 11.20 | 10.30 | 11.50 | 9.70 | 14.10 | 9.70 | 14.70 | 10.90 |
| Lithuania DBD Rate | - | - | 7.90 | 11.20 | 10.30 | 11.50 | 9.70 | 14.10 | 9.70 | 14.70 | 10.90 |
| Lithuania DCD Rate | - | - | - | - | - | - | - | - | - | - | - |
| Luxembourg DD Rate | 17.50 | 12.50 | 7.50 | - | 2.10 | 6.30 | 12.70 | 2.10 | 18.40 | - | 6.02 |
| Luxembourg DBD Rate | 17.50 | 12.50 | 7.50 | - | 2.10 | 6.30 | 12.70 | 2.10 | 18.40 | - | 6.02 |
| Luxembourg DCD Rate | - | - | - | - | - | - | - | - | - | - | - |
| Poland DD Rate | 10.60 | 11.60 | 12.60 | 13.70 | 14.50 | 14.50 | 13.00 | 9.20 | 11.20 | 11.00 | 13.30 |
| Poland DBD Rate | 10.60 | 11.60 | 12.60 | 13.70 | 14.50 | 14.50 | 13.00 | 9.20 | 11.20 | 11.00 | 13.30 |
| Poland DCD Rate | - | - | - | - | - | - | - | - | - | - | - |
| Costa Rica DD Rate | - | 10.70 | 10.00 | 5.80 | 11.00 | 10.20 | 11.30 | 7.70 | - | - | - |
| Costa Rica DBD Rate | - | 10.70 | 10.00 | 5.80 | 11.00 | 10.20 | 11.30 | 7.70 | - | - | - |
| Costa Rica DCD Rate | - | 0.00 | 0.00 | 0.00 | 0.00 | 0.00 | 0.00 | 0.00 | - | - | - |
