# Supplementary material for: An International Comparison of the Effect of Policy Shifts to Organ Donation following Cardiocirculatory Death (DCD) on Donation Rates after Brain Death (DBD) and Transplantation Rates
Source: PLoS One. 2013 May 7;8(5):e62010. doi: 10.1371/journal.pone.0062010 (PMC3647074; doi:10.1371/journal.pone.0062010)
Supplement: Appendix S4 — (DOCX) [file pone.0062010.s004.docx]

**Appendix S4**

**Group Three Countries (10 DD pmp per year) – DD, DBD & DCD Rates in pmp**

| **Country** | **2000** | **2001** | **2002** | **2003** | **2004** | **2005** | **2006** | **2007** | **2008** | **2009** | **2010** |
| --- | --- | --- | --- | --- | --- | --- | --- | --- | --- | --- | --- |
| Iceland DD Rate | - | - | - | 0.00 | 17.10 | 6.80 | 19.50 | 0.00 | 6.30 | 18.80 | 9.44 |
| Iceland DBD Rate | - | - | - | 0.00 | 17.10 | 6.80 | 19.50 | 0.00 | 6.30 | 18.80 | 9.44 |
| Iceland DCD Rate | - | - | - | 0.00 | 0.00 | 0.00 | 0.00 | 0.00 | 0.00 | 0.00 | 0.00 |
| Cyprus DD Rate | - | 3.0 | 10.70 | 1.40 | 11.40 | 7.10 | 5.70 | 12.86 | 19.00 | - | - |
| Cyprus DBD Rate | - | 3.00 | 10.70 | 1.40 | 11.40 | 7.10 | 5.70 | 12.86 | 19.00 | - | - |
| Cyprus DCD Rate | - | - | - | - | - | - | - | - | - | - | - |
| Colombia DD Rate | - | - | 4.20 | - | - | 6.90 | 9.90 | 10.70 | 9.60 | 12.30 | 12.50 |
| Colombia DBD Rate | - | - | 4.20 | - | - | 6.90 | 9.90 | 10.70 | 9.60 | 10.90 | 12.50 |
| Colombia DCD Rate | - | - | 0.00 | - | - | 0.00 | 0.00 | 0.00 | 0.00 | 1.60 | 0.00 |
| Israel DD Rate | 12.00 | 9.50 | 9.80 | 6.40 | 8.80 | 8.90 | 9.80 | 7.70 | 9.80 | 8.80 | - |
| Israel DBD Rate | 12.00 | 9.50 | 9.80 | 6.40 | 8.70 | 8.50 | 9.70 | 7.70 | 9.80 | 8.67 | - |
| Israel DCD Rate | 0.00 | 0.00 | 0.00 | 0.00 | 0.10 | 0.40 | 0.00 | 0.00 | 0.00 | 0.13 | - |
| New Zealand DD Rate | 10.60 | 9.50 | 9.60 | 9.90 | 9.80 | 7.00 | 6.00 | 8.90 | 7.30 | 9.96 | 9.40 |
| New Zealand DBD Rate | 10.60 | 9.50 | 9.60 | 9.90 | 9.80 | 7.00 | 6.00 | 8.90 | 6.80 | 9.46 | 9.20 |
| New Zealand DCD Rate | 0.00 | 0.00 | 0.00 | 0.00 | 0.00 | 0.00 | 0.00 | 0.00 | 0.50 | 0.50 | 0.20 |
| Chile DD Rate | 9.70 | 8.30 | 7.50 | 8.60 | 8.90 | 8.20 | 10.00 | 8.10 | 7.10 | - | - |
| Chile DBD Rate | 9.70 | 8.30 | 7.50 | 8.60 | 8.90 | 8.20 | 10.00 | 8.10 | 7.10 | - | - |
| Chile DCD Rate | 0.00 | 0.00 | 0.00 | 0.00 | 0.00 | 0.00 | 0.00 | 0.00 | 0.00 | - | - |
| Brazil DD Rate | 4.20 | 4.30 | 5.30 | 6.60 | 7.30 | 6.30 | 6.00 | 6.20 | 7.00 | 8.00 | 9.60 |
| Brazil DBD Rate | 4.20 | 4.30 | 5.30 | 6.60 | 7.30 | 6.30 | 6.00 | 6.20 | 7.00 | 8.00 | 9.60 |
| Brazil DCD Rate | 0.00 | 0.00 | 0.00 | 0.00 | 0.00 | 0.00 | 0.00 | 0.00 | 0.00 | 0.00 | 0.00 |
| Greece DD Rate | 1.90 | - | 5.90 | 6.30 | 6.10 | 8.10 | 7.20 | 5.80 | 8.90 | 6.50 | - |
| Greece DBD Rate | 1.90 | - | 5.90 | 6.30 | 6.10 | 8.10 | 7.20 | 5.80 | 8.90 | 6.50 | - |
| Greece DCD Rate | 0.00 | - | 0.00 | 0.00 | 0.00 | 0.00 | 0.00 | 0.00 | 0.00 | 0.00 | -- |
| Taiwan DD Rate | 2.40 | - | - | - | - | - | 7.20 | 6.60 | 8.50 | - | - |
| Taiwan DBD Rate | 2.40 | - | - | - | - | - | 7.20 | 6.60 | 8.50 | - | - |
| Taiwan DCD Rate | 0.00 | - | - | - | - | - | 0.00 | 0.00 | 0.00 | - | - |
| Hong Kong SARC DD Rate | 4.00 | 4.50 | 6.40 | 3.80 | 4.20 | 4.20 | 4.30 | 4.70 | 5.30 | 7.54 | 6.70 |
| Hong Kong SARC DBD Rate | 4.00 | 4.50 | 6.40 | 3.80 | 4.20 | 4.20 | 4.30 | 4.70 | 5.30 | 7.54 | 6.70 |
| Hong Kong SARC DCD Rate | 0.00 | 0.00 | 0.00 | 0.00 | 0.00 | 0.00 | 0.00 | 0.00 | 0.00 | 0.00 | 0.00 |
| Singapore DD Rate | - | 7.90 | 4.70 | - | 4.80 | 5.90 | - | - | - | 4.60 | 5.10 |
| Singapore DBD Rate | - | 7.90 | 4.70 | - | 4.52 | 5.62 | - | - | - | 4.00 | 4.51 |
| Singapore DCD Rate | - | 0.00 | 0.00 | - | 0.28 | 0.28 | - | - | - | 0.60 | 0.59 |
| Saudi Arabia DD Rate | 1.80 | 2.70 | 1.90 | 2.10 | 2.60 | 2.90 | 4.70 | 3.50 | 4.50 | 3.31 | 3.86 |
| Saudi Arabia DBD Rate | 1.80 | 2.70 | 1.90 | 2.10 | 2.60 | 2.90 | 4.70 | 3.50 | 4.40 | 3.31 | 3.86 |
| Saudi Arabia DCD Rate | 0.00 | 0.00 | 0.00 | 0.00 | 0.00 | 0.00 | 0.00 | 0.00 | 0.10 | 0.00 | 0.00 |
| Qatar DD Rate | - | - | 1.30 | 2.60 | 6.60 | 5.30 | 4.00 | 4.00 | 4.00 | 2.60 | - |
| Qatar DBD Rate | - | - | 1.30 | 2.60 | 6.60 | 5.30 | 4.00 | 4.00 | 4.00 | 2.60 | - |
| Qatar DCD Rate | - | - | 0.00 | 0.00 | 0.00 | 0.00 | 0.00 | 0.00 | 0.00 | 0.00 | - |
| Kuwait DD Rate | - | - | - | - | 6.40 | 5.90 | - | - | - | - | - |
| Kuwait DBD Rate | - | - | - | - | 6.40 | 5.90 | - | - | - | - | - |
| Kuwait DCD Rate | - | - | - | - | 0.00 | 0.00 | - | - | - | - | - |
| Panama DD Rate | 1.00 | 3.20 | 5.00 | 5.30 | 6.30 | 3.30 | - | - | - | - | - |
| Panama DBD Rate | 1.00 | 3.20 | 5.00 | 5.30 | 6.30 | 3.30 | - | - | - | - | - |
| Panama DCD Rate | 0.00 | 0.00 | 0.00 | 0.00 | 0.00 | 0.00 | - | - | - | - | - |
| Turkey DD Rate | 0.70 | 1.30 | 1.40 | 1.50 | 2.00 | 2.30 | 2.50 | 3.00 | 3.60 | 4.20 | 3.50 |
| Turkey DBD Rate | 0.70 | 1.30 | 1.40 | 1.50 | 2.00 | 2.30 | 2.50 | 3.00 | 3.56 | 4.20 | 3.50 |
| Turkey DCD Rate | 0.00 | 0.00 | 0.00 | 0.00 | 0.00 | 0.00 | 0.00 | 0.00 | 0.04 | 0.00 | 0.00 |
| South Africa DD Rate | 3.10 | 3.00 | 2.50 | 2.00 | - | 4.50 | - | - | - | - | - |
| South Africa DBD Rate | 3.10 | 3.00 | 2.50 | 2.00 | - | 4.50 | - | - | - | - | - |
| South Africa DCD Rate | 0.00 | 0.00 | 0.00 | 0.00 | - | 0.00 | - | - | - | - | - |
| Iran DD Rate | 0.20 | 0.60 | 0.70 | 1.20 | 1.60 | 1.70 | 1.80 | 2.30 | 2.90 | 2.90 | 4.10 |
| Iran DBD Rate | 0.20 | 0.60 | 0.70 | 1.20 | 1.60 | 1.70 | 1.80 | 2.30 | 2.90 | 2.90 | 4.10 |
| Iran DCD Rate | 0.00 | 0.00 | 0.00 | 0.00 | 0.00 | 0.00 | 0.00 | 0.00 | 0.00 | 0.00 | 0.00 |
| Venezuela DD Rate | 1.60 | 2.10 | 2.60 | 1.80 | 1.90 | 1.60 | 2.30 | 3.70 | 3.30 | 3.20 | 3.50 |
| Venezuela DBD Rate | 1.60 | 2.10 | 2.60 | 1.80 | 1.90 | 1.60 | 2.30 | 3.70 | 3.30 | 3.20 | 3.50 |
| Venezuela DCD Rate | 0.00 | 0.00 | 0.00 | 0.00 | 0.00 | 0.00 | 0.00 | 0.00 | 0.00 | 0.00 | 0.00 |
| Mexico DD Rate | - | - | - | - | - | 2.90 | 3.00 | 3.23 | 3.10 | 2.90 | 2.00 |
| Mexico DBD Rate | - | - | - | - | - | 2.90 | 3.00 | 3.23 | 3.10 | 2.90 | 2.00 |
| Mexico DCD Rate | - | - | - | - | - | 0.00 | 0.00 | 0.00 | 0.00 | 0.00 | 0.00 |
| Bahrain DD Rate | - | - | - | - | 0.00 | 4.00 | - | - | - | - | - |
| Bahrain DBD Rate | - | - | - | - | 0.00 | 4.00 | - | - | - | - | - |
| Bahrain DCD Rate | - | - | - | - | 0.00 | 0.00 | - | - | - | - | - |
| Romania DD Rate | 1.00 | 0.90 | 0.60 | 0.30 | 0.40 | 0.50 | 1.00 | 1.71 | 2.85 | 2.00 | 3.18 |
| Romania DBD Rate | 1.00 | 0.90 | 0.60 | 0.30 | 0.40 | 0.50 | 1.00 | 1.71 | 2.85 | 2.00 | 3.18 |
| Romania DCD Rate | 0.00 | 0.00 | 0.00 | 0.00 | 0.00 | 0.00 | 0.00 | 0.00 | 0.00 | 0.00 | 0.00 |
| Peru DD Rate | - | 2.60 | 1.30 | - | - | - | - | - | - | - | 3.20 |
| Peru DBD Rate | - | 2.60 | 1.30 | - | - | - | - | - | - | - | 3.20 |
| Peru DCD Rate | - | 0.00 | 0.00 | - | - | - | - | - | - | - | 0.00 |
| South Korea DD Rate | - | - | - | - | - | 1.90 | 2.80 | 2.97 | - | - | - |
| South Korea DBD Rate | - | - | - | - | - | 1.90 | 2.80 | 2.97 | - | - | - |
| South Korea DCD Rate | - | - | - | - | - | 0.00 | 0.00 | 0.08 | - | - | - |
| Russia DD Rate | - | - | - | - | - | - | - | - | 2.70 | 270 | 3.40 |
| Russia DBD Rate | - | - | - | - | - | - | - | - | 1.40 | 1.40 | 2.00 |
| Russia DCD Rate | - | - | - | - | - | - | - | - | 1.30 | 1.30 | 1.40 |
| Bulgaria DD Rate | - | - | - | - | - | 1.90 | 2.80 | 1.20 | 0.80 | 1.60 | 2.85 |
| Bulgaria DBD Rate | - | - | - | - | - | 1.90 | 2.80 | 1.20 | 0.80 | 1.60 | 2.85 |
| Bulgaria DCD Rate | - | - | - | - | - | 0.00 | 0.00 | 0.00 | 0.00 | 0.00 | 0.00 |
| Bolivia DD Rate | - | - | - | - | - | - | - | - | 2.00 | - | 1.40 |
| Bolivia DBD Rate | - | - | - | - | - | - | - | - | 2.00 | - | 1.40 |
| Bolivia DCD Rate | - | - | - | - | - | - | - | - | 0.00 | - | 0.00 |
| Ecuador DD Rate | - | - | - | - | 1.00 | 2.40 | - | - | - | 1.20 | 2.50 |
| Ecuador DBD Rate | - | - | - | - | 1.00 | 2.40 | - | - | - | 1.20 | 2.50 |
| Ecuador DCD Rate | - | - | - | - | 0.00 | 0.00 | - | - | - | 0.00 | 0.00 |
| Lebanon DD Rate | - | - | - | 2.50 | 0.70 | 0.00 | 1.30 | - | 0.50 | - | 0.50 |
| Lebanon DBD Rate | - | - | - | 2.50 | 0.70 | 0.00 | 1.30 | - | 0.50 | - | 0.50 |
| Lebanon DCD Rate | - | - | - | 0.00 | 0.00 | 0.00 | 0.00 | - | 0.00 | - | 0.00 |
| Paraguay DD Rate | - | - | - | - | - | - | - | - | - | - | 1.90 |
| Paraguay DBD Rate | - | - | - | - | - | - | - | - | - | - | 1.90 |
| Paraguay DCD Rate | - | - | - | - | - | - | - | - | - | - | 0.00 |
| Tunisia DD Rate | - | - | - | - | - | - | - | - | 1.40 | - | - |
| Tunisia DBD Rate | - | - | - | - | - | - | - | - | 1.40 | - | - |
| Tunisia DCD Rate | - | - | - | - | - | - | - | - | 0.00 | - | - |
| Japan DD Rate | - | 0.60 | 0.50 | 0.60 | 0.80 | 0.70 | 0.90 | 0.80 | 0.90 | 0.80 | 0.90 |
| Japan DBD Rate | - | 0.00 | 0.00 | 0.00 | 0.10 | 0.00 | 0.10 | 0.10 | 0.10 | 0.00 | 0.30 |
| Japan DCD Rate | - | 0.60 | 0.50 | 0.60 | 0.70 | 0.70 | 0.80 | 0.70 | 0.80 | 0.80 | 0.60 |
| Moldova DD Rate | - | 1.00 | 0.90 | 0.90 | - | 0.00 | 0.00 | 0.00 | - | - | - |
| Moldova DBD Rate | - | 1.00 | 0.90 | 0.90 | - | 0.00 | 0.00 | 0.00 | - | - | - |
| Moldova DCD Rate | - | 0.00 | 0.00 | 0.00 | - | 0.00 | 0.00 | 0.00 | - | - | - |
| Malaysia DD Rate | - | 0.80 | 0.50 | 0.30 | 0.30 | 0.20 | 0.50 | 0.50 | 0.50 | 0.70 | 0.70 |
| Malaysia DBD Rate | - | 0.80 | 0.50 | 0.30 | 0.30 | 0.20 | 0.50 | 0.50 | 0.50 | 0.70 | 0.70 |
| Malaysia DCD Rate | - | 0.00 | 0.00 | 0.00 | 0.00 | 0.00 | 0.00 | 0.00 | 0.00 | 0.00 | 0.00 |
| Dominican Republic DD Rate | - | - | - | - | 0.00 | 0.00 | - | - | 0.80 | - | - |
| Dominican Republic DBD Rate | - | - | - | - | 0.00 | 0.00 | - | - | 0.80 | - | - |
| Dominican Republic DCD Rate | - | - | - | - | 0.00 | 0.00 | - | - | 0.00 | - | - |
| Ukraine DD Rate | - | - | - | 0.40 | 0.30 | 0.30 | 0.50 | 0.60 | 0.30 | 0.50 | - |
| Ukraine DBD Rate | - | - | - | 0.40 | 0.30 | 0.30 | 0.50 | 0.60 | 0.30 | 0.50 | - |
| Ukraine DCD Rate | - | - | - | 000 | 0.00 | 0.00 | 0.00 | 0.00 | 0.00 | 0.00 | - |
| Guatemala DD Rate | - | - | - | 0.30 | 0.10 | - | 0.20 | - | - | - | - |
| Guatemala DBD Rate | - | - | - | 0.30 | 0.10 | - | 0.20 | - | - | - | - |
| Guatemala DCD Rate | - | - | - | 0.00 | 0.00 | - | 0.00 | - | - | - | - |
| Trinidad & Tobago DD Rate | - | - | - | - | - | - | 0.00 | 0.80 | - | - | 0.00 |
| Trinidad & Tobago DBD Rate | - | - | - | - | - | - | 0.00 | 0.80 | - | - | 0.00 |
| Trinidad & Tobago DCD Rate | - | - | - | - | - | - | 0.00 | 0.00 | - | - | 0.00 |
| Jordan DD Rate | - | - | - | 0.30 | 0.00 | 0.00 | 0.00 | - | - | - | - |
| Jordan DBD Rate | - | - | - | 0.30 | 0.00 | 0.00 | 0.00 | - | - | - | - |
| Jordan DCD Rate | - | - | - | 0.00 | 0.00 | 0.00 | 0.00 | - | - | - | - |
